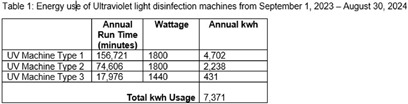# Assessing the Impact of Ultraviolet Light Disinfection on Energy Use at Stanford Health Care

**DOI:** 10.1017/ash.2025.326

**Published:** 2025-09-24

**Authors:** Mindy Sampson, Angel Do, Paige Fox, Eugenia Miranti, Guillermo Rodriguez Nava, Karen McIntyre, Inna Dolottseva, Erika Kimball, Jorge Salinas

**Affiliations:** 1 Stanford University; 2 Stanford Health Care; 3 Stanford University School of Medicine; 4 Stanford Health Care; 5 Stanford Healthcare

## Abstract

**Introduction:** The healthcare sector contributes significantly to global greenhouse gas (GHG) emissions, accounting for 8.5% of the total emissions in the United States alone. Infection control practices designed to prevent disease transmission contribute to this substantial carbon footprint. These practices, which include enhanced ventilation requirements, extensive sterilization processes, and laundry services, inherently increase energy consumption and GHG emissions. The approach to making healthcare more sustainable has been multipronged, including initiatives to reduce waste and energy use. We explored environmental cleaning practices and the energy use associated with ultraviolent (UV) light disinfection. **Methods:** A retrospective analysis was conducted on the energy consumption of three different UV light disinfection machines used at Stanford Health Care from September 2023 to August 2024. Annual run time data was obtained from vendor-provided logs, and energy use was calculated using the equipment wattage specifications provided with each machine. **Results:** We found that UV light disinfection utilized approximately 7,300 kWh constituting less than 1% of Stanford Health Care total energy use for the period. This energy consumption equates to the charge required for 3.5 round trips from San Francisco to New York City in an electric vehicle. **Discussion:** UV light has been widely used in healthcare over the last decade. Recent data suggests that there may be no additional benefit to UV light disinfection when other enhanced cleaning methods, such as sporicidal cleaners, are utilized. Therefore, using UV light in addition to sporicidal cleaners may be redundant. Infection prevention practices often incorporate redundancies given the dependence on human behavior and the high consequences of practice failures. As the healthcare industry continues to work towards reducing greenhouse gas emissions it will be important to consider all energy reductions and any redundancies in practices. In the future, a life cycle analysis could be conducted to compare UV light disinfection and sporicidal cleaning methods to evaluate each practice’s impact on sustainability efforts. Our evaluation showed that UV light disinfection results in modest energy usage reductions. However, as the healthcare industry continues to work towards reducing greenhouse gas emissions it will be important to consider all energy reductions and any redundancies in practices.

**Figure:**